# Harnessing Plasmon-Induced Hot Carriers at the Interfaces With Ferroelectrics

**DOI:** 10.3389/fchem.2019.00299

**Published:** 2019-05-14

**Authors:** Vineet Kumar, Shaun C. O'Donnell, Daniel L. Sang, Paul A. Maggard, Gufeng Wang

**Affiliations:** Department of Chemistry, North Carolina State University, Raleigh, NC, United States

**Keywords:** ferroelectrics, surface plasmon resonance, Schottky barrier, spontaneous polarization, hot electrons, charge injection, photocatalysis

## Abstract

This article reviews the scientific understanding and progress of interfacing plasmonic particles with ferroelectrics in order to facilitate the absorption of low-energy photons and their conversion to chemical fuels. The fundamental principles of hot carrier generation and charge injection are described for semiconductors interfaced with metallic nanoparticles and immersed in aqueous solutions, forming a synergistic juncture between the growing fields of plasmonically-driven photochemistry and semiconductor photocatalysis. The underlying mechanistic advantages of a metal-ferroelectric vs. metal-nonferroelectric interface are presented with respect to achieving a more optimal and efficient control over the Schottky barrier height and charge separation. Notable recent examples of using ferroelectric-interfaced plasmonic particles have demonstrated their roles in yielding significantly enhanced photocurrents as well as in the photon-driven production of molecular hydrogen. Notably, plasmonically-driven photocatalysis has been shown to occur for photon wavelengths in the infrared range, which is at lower energies than typically possible for conventional semiconductor photocatalysts. Recent results thus demonstrate that integrated ferroelectric-plasmonic systems represent a potentially transformative concept for use in the field of solar energy conversion.

## Background

### Generation of Hot Carriers in Plasmonic Nanoparticles

**Figure 1:**
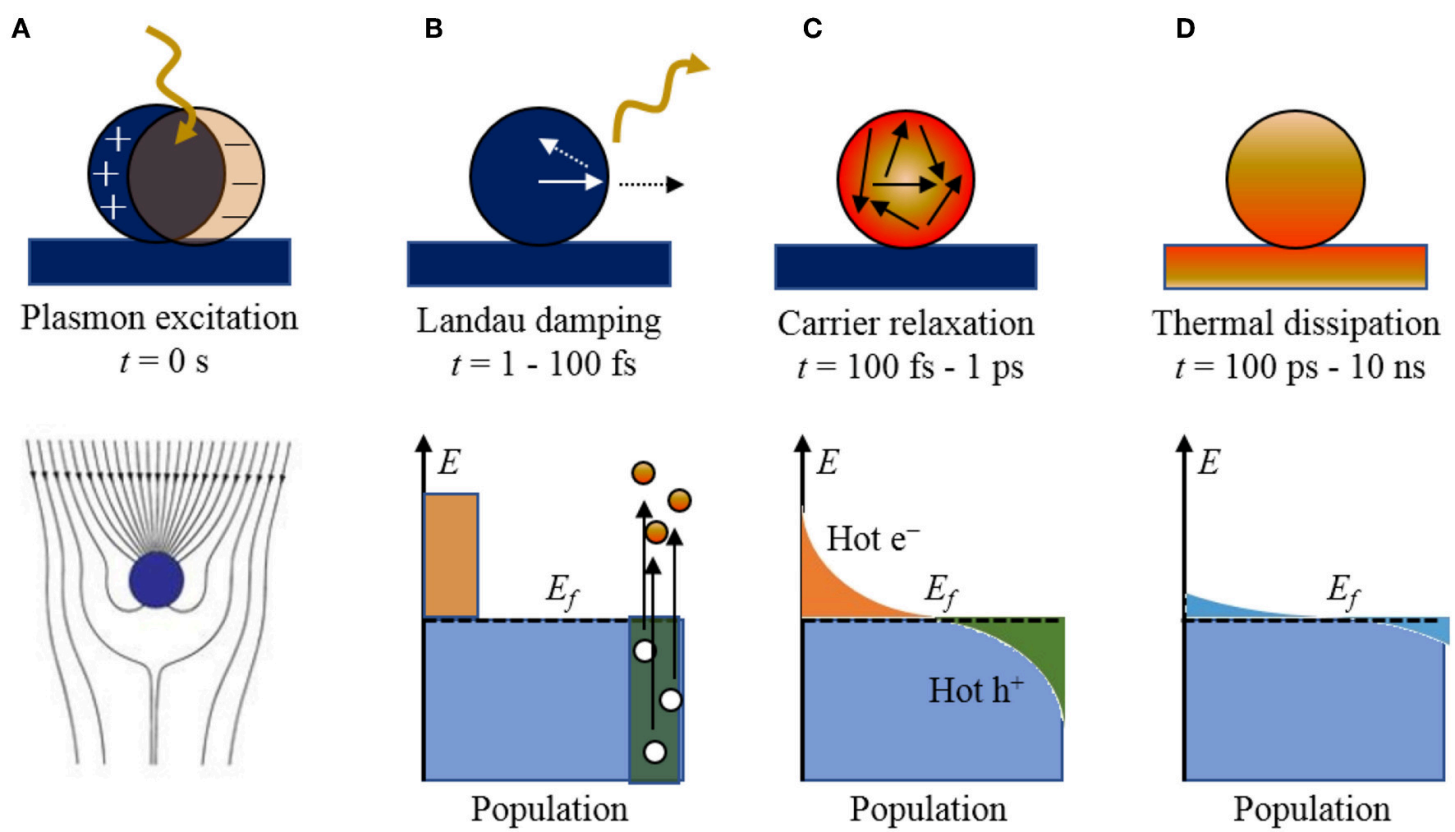
The stepwise processes of photoexcitation and electronic relaxation for a metallic nanoparticle with the characteristic timescales and population of electronic states: **(A)** excitation of a localized surface plasmon, **(B)** Landau damping and the creation of electron-hole pairs, **(C)** carrier relaxation and thermalized “hot” distribution of carriers, and **(D)** thermal dissipation to the surroundings. The Fermi level is labeled by *E*_*f*_. Adapted with permission from Brongersma et al. (2015). Copyright 2015 Spring Nature.

The transformation of solar energy into chemical potential energy, e.g., such that which is stored in molecular hydrogen, represents a sustainable and favorable pathway to eventually displacing fossil fuels as the predominant energy resource. Noble metals have been drawing increasing attention as a component of efficient light-harvesting systems arising from the collective oscillation of their conduction electrons, i.e., surface plasmon resonances (SPR), that can be excited by photons over a very wide range of infrared to ultraviolet wavelengths (Brongersma et al., 2015; Linic et al., 2015; Moskovits, 2015). Within a metallic nanoparticle, localized surface plasmons are produced that are confined to an area that is similar to or smaller than the wavelength of light excitation. Subsequent to the absorption of light, a number of competing elementary processes and potential outcomes can occur (Figure 1), with timescales that are dependent upon the nanoparticle size, shape, and electronic structure. The basic features of these competing processes have been the focus of reviews.

Briefly, when a metal particle is irradiated by light, the electromagnetic field induces the polarization of the conduction electrons with respect to the positive ionic cores, creating an electric dipole (Figure 1A). The Columbic interaction between the opposite charges acts as a restoring force, which leads to electronic oscillations, where the collective electronic oscillations are named plasmons. When the electronic oscillations occur locally at the nanoparticle as shown in Figure 1A, the corresponding surface oscillation is known as localized SPR (LSPR). Under resonance conditions, the particle absorption cross section is greatly enhanced, e.g., up to five orders of magnitude larger than those of typical dye molecules (Jain et al., 2006). The SPR wavelengths of the plasmonic nanoparticles are tunable, by varying size and shape, across the entire visible spectrum. The confined electronic oscillations in the particle enhance the local electric field distribution at the surface of the nanostructures. This leads to several interesting phenomena such as Surface-Enhanced Raman Scattering (SERS), etc., which is beyond the scope of this review article.

The plasmon resonances can decay radiatively by re-emission of photons or non-radiatively by Landau damping with the creation of electron-hole pairs, on the timescale of ~1–100 fs. For small nanoparticles (~10–25 nm) and sub-radiant plasmon modes, the branching ratio is predominated by the creation of electron-hole pairs. An example of the non-thermalized distribution of charged carriers is illustrated in Figure 1B, showing “hot” electrons and holes that are energetically above and below the Fermi level of the metal particle, respectively (Mukherjee et al., 2013; Linic et al., 2015). The resulting distribution is a sensitive function of the plasmon energy, particle size, and the electronic structure of the metallic nanoparticle, as described previously. These hot carriers will adiabatically re-equilibrate via electron scattering and Auger transitions on the order of 100 fs to 1 ps (Figure 1C) to produce a Fermi-Dirac like distribution with a high effective electron temperature of several thousand degrees. Thus, *high energy hot electrons* can be produced in the process even when the particle is excited by relatively low energy photons (Moskovits, 2015).

**Figure 2:**
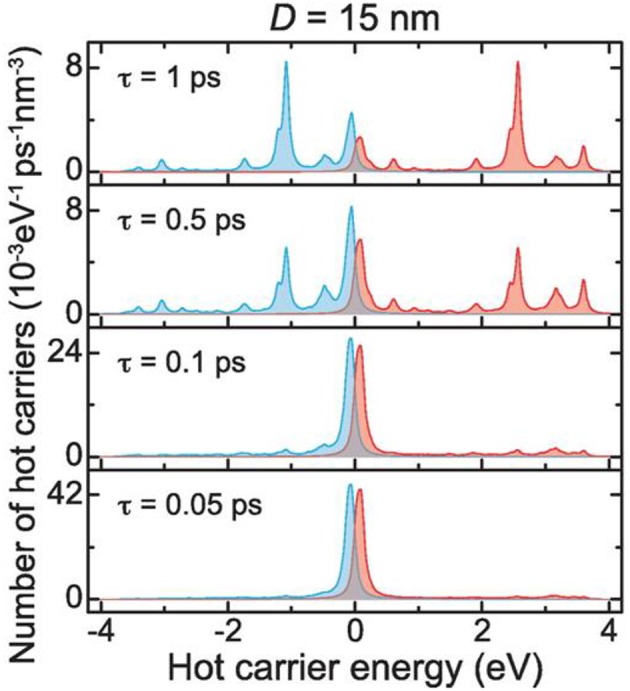
Hot carrier distributions per unit time and volume as a function of energy, calculated for a 15 nm diameter Ag nanoparticle. Hot electrons and hot holes are plotted as the blue and red lines, respectively, with the Fermi level set at the zero energy. The stacked plots show the changes in the hot carrier distributions for relaxation lifetimes from 0.05 to 1.0 ps. An incident photon energy of 3.65 eV was used. Adapted with permission from Manjavacas et al. (2014). Copyright 2014 American Chemical Society.

Shown in Figure 2, for example, a recent investigation into 15 nm Ag nanoparticles shows a wide calculated distribution of hot carriers for a continuously-irradiated particle and lifetimes that varied between 0.05 and 1.0 ps. Thus, the energetic distribution of the hot carriers, i.e., both their densities and energies, is a highly sensitive function of their re-equilibration lifetimes. For a photon energy of 3.65 eV, a wide range of excited states is ultimately achieved that ranges from 3 to 4 eV both above and below the Fermi level for the hot electron and hot hole states, respectively, as shown in Figure 2. These represent electrochemical potentials for both electrons and holes that exceed the band energy requirements for the water-splitting half reactions, as will be described below. For a larger particle size of 25 nm, a significantly smaller hot-carrier density (~50%) results because of the higher number of allowable electronic states. Lastly, the thermal re-equilibration with the lattice requires on the order of several picoseconds (Figure 1D), followed by heat transfer to the surroundings in ~10 ps to 10 ns.

Critical to solar energy conversion, high-energy hot electrons and holes are generated in plasmonic nanoparticles during the light absorption processes, with lifetimes on the order of 10 ps before thermal equilibration with the surroundings (Hoggard et al., 2013; Major et al., 2014; Linic et al., 2015). This realization has led to a surge of recent studies that have demonstrated that the excitation of the SPR of particle-based catalysts can be used to facilitate or greatly amplify their catalytic activities (Adleman et al., 2009; Bueno Alejo et al., 2011; Christopher et al., 2011; Liu et al., 2011; Warren and Thimsen, 2012; Hoggard et al., 2013; Mubeen et al., 2013; Mukherjee et al., 2013; Major et al., 2014; Marchuk and Willets, 2014; Linic et al., 2015). How specific catalytic reactions are impacted by excitation of the plasmonic nanoparticle is still under intense investigation and likely varies case-by-case. Generally, it is viewed that SPR can enhance photon-driven chemical reactions through two possible mechanisms. These include the local photothermal effect, i.e., the reaction is thermally activated or expedited by the hot lattice produced by thermal re-equilibration, and more interestingly, by hot charge transfer across interfaces to molecular absorbates to electrochemically induce reactivity. In the latter pathway, the excitation of the SPR causes the formation of hot electron-hole pairs that can then be transferred to the metal surface and the adsorbate molecules directly or indirectly, inducing the chemical reactions (Major et al., 2014; Brandt et al., 2016).

Prominent early examples of hot electron transfer include the dissociation of H_2_ molecules on the surface of ~10–20 nm Au particles, or the oxidation of ethylene by O_2_ using 60 nm Ag nanocubes (Mukherjee et al., 2014). For the room-temperature dissociation of H_2_ molecules, the study found that hot electrons transferred most effectively across a nanoshell of SiO_2_ and less effectively across a nanoshell of TiO_2_, owing to the lower-energy conduction band of TiO_2_ that functions as a highly effective electron acceptor. Conversely, hot hole transfer has been demonstrated in the growth of Ag nanoprisms via oxidation of citrate anions in solution (Jin et al., 2003). The plasmon-induced growth of the Ag nanoprisms was found to be more sensitively dependent on the incident photon energy rather than the photon flux, owing to the increasing oxidizing power of the hot holes. Many other remarkable studies in this field have proven that the energies and lifetimes of the hot electron and holes can be sensitively manipulated and used to enable their facile transfer to adsorbates and/or through interfaces. These can be utilized to drive reactions that have significant activation energies, or alternatively, reactions that are thermodynamically uphill in energy.

### Light-Driven Reactivity at Semiconductor Surfaces

The realization that light-absorption by semiconductors could be used to drive chemical reactions occurred in the early 1970s with the seminal research on *n*-type TiO_2_ that demonstrated that water could be oxidized at its surfaces to molecular oxygen (Fujishima and Honda, 1972; Kawai and Sakata, 1980; Hoffmann et al., 1995; Kato et al., 2003; Maeda et al., 2005; Linic et al., 2011; Sivula et al., 2011; Ohno et al., 2012; Huang et al., 2017; Suzuki et al., 2018). Since that time, semiconductors from among many different chemical systems have been investigated for their ability to function at high solar-to-chemical efficiencies and stabilities, including in the oxides, chalcogenides, and nitrides. These have included in applications such as in thin-film photovoltaics and photoelectrodes, as well as in the form of photocatalyst particles. However, the anatase polymorph of TiO_2_ has been among the most highly investigated materials for the water splitting reactions to produce molecular hydrogen and oxygen (Kawai and Sakata, 1980; Hoffmann et al., 1995).

**Figure 3:**
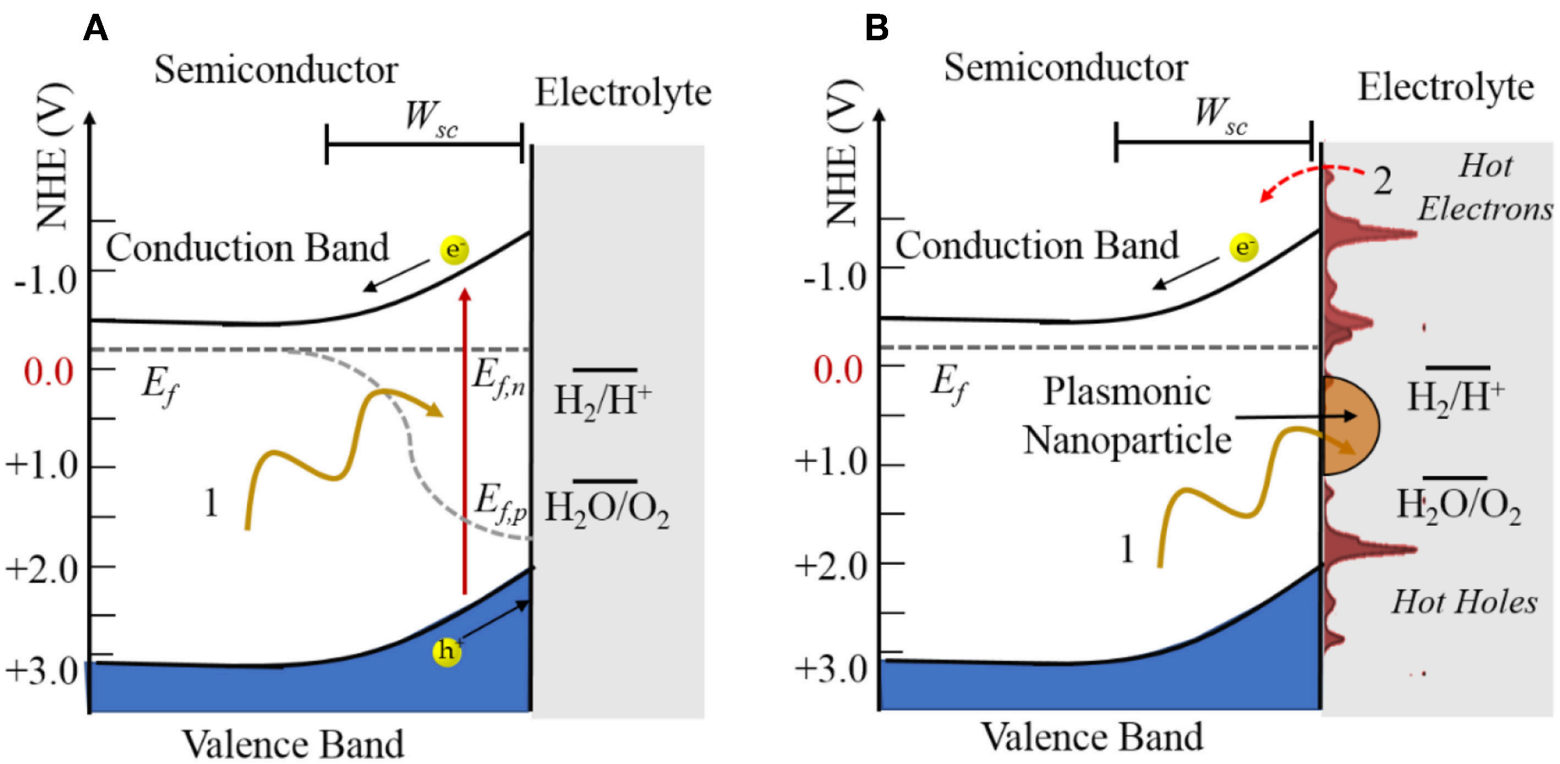
Illustration of **(A)** an *n*-type semiconductor-electrolyte interface under irradiation and **(B)** an *n*-type semiconductor with a plasmonic nanoparticle attached at the surface. The quasi-Fermi levels in **(A)** are labeled by *E*_*f,n*_ and *E*_*f,p*_ and the Fermi level in **(B)** labeled by *E*_*f*_. The space charge width is labeled by *W*_*sc*_.

Shown in Figure 3A, a photocatalytic reaction consists of three basic steps, including the absorption of bandgap photons with the generation of electron (e^−^) and hole (h^+^) pairs, charge separation and transport to the surfaces via their diffusion and drift, and then electron transfer at the surfaces to drive redox reactions, e.g., such as for water splitting. A key element to the charge separation is the space charge layer that forms at the semiconductor surface as a result of its equilibration with the chemical potential of the solution. In an *n*-type semiconductor, this results in an upward band bending with a depth given by *W*_*sc*_ that is a function of the dopant density, dielectric constant, and space charge height. The electron-hole pairs will be separated if they are produced within this layer or if they can diffuse to it before recombining. In this case, the oxidation reaction occurs at the semiconductor surface and the reduction reaction occurs at a counter-electrode or at a surface cocatalyst island such as Pt.

The production of molecular hydrogen from the splitting of water is a thermodynamically uphill process with a Gibbs' free energy change of ~238 KJ/mol, thus requiring the equivalent of >1.23 eV (<1,008 nm) per photon (Maeda et al., 2006; Ingram and Linic, 2011; Sivula et al., 2011; Wang and Astruc, 2014). A semiconductor with a bandgap size of >1.23 eV and suitable band energies that straddle the water redox couples can be used to drive the water splitting reaction at its surfaces from solar irradiation. A consideration of the required overpotentials at its surface catalyst sites increases the optimal photon energy to ~1.6–2.0 eV, or to wavelengths less than about ~600–775 nm for a single junction photoelectrode or photocatalyst particle. Considering that the solar irradiation, i.e., AM 1.5 G light, includes a large (>50%) contribution in the infrared energy range with wavelengths of >800 nm, there is a significant fraction of solar energy that cannot be utilized in single-junction semiconductor systems for photocatalysis (Navarro et al., 2009; Long et al., 2015). Other factors that negatively impact the light-driven reactions at the surfaces include the efficiency of charge separation, charge carrier lifetimes, and surface reaction rates and stability. Owing to these limitations, their practical efficiency in converting sunlight to chemical energy remains low (<1% for total solar energy) compared to the minimum solar-to-hydrogen efficiency of ~10% that is required for commercial viability (Lewerenz and Peter, 2013).

Under ultraviolet irradiation, many large bandgap semiconductors show a high quantum efficiency and outstanding stability, e.g., La-doped NaTaO_3_ and TiO_2_ with bandgap sizes of ~3–4.5 eV. This has led to numerous studies aimed at their sensitization to visible-light wavelengths, such as using molecular dyes via charge injection of their excited electrons. However, most molecular dyes degrade rapidly over time or cannot be used to drive the overall water splitting reaction. Plasmonic nanoparticles, either bare or combined with a semiconductor, have most recently been the subject of several key investigations for use in light-driven reactions (Zhu, 1994; Wang et al., 2008; Li et al., 2015; Ma, X. et al., 2016; Ortiz et al., 2017). The placement of a plasmonic particle and a semiconductor surface in close vicinity can have a remarkable impact on their catalytic activity. For example, Ma et al. found that the photocatalytic activity in a combined plasmonic-semiconductor particle can be increased through local electric field enhancement of photo-induced charge carrier generation at the semiconductor surface (Ma et al., 2014a). The photo-degradation of methyl orange under visible-light irradiation was investigated for Ag@AgCl, where AgCl is a wide band gap material (>3 eV). It was proposed that energy transfer from the Ag nanoparticle to the semiconductor AgCl required the presence of mid-gap defect states. In this case, the SPR of the Ag nanoparticles was found to enhance the excitations of AgCl involving these defect states.

More generally, photogenerated hot charge carrier injection from the plasmonic particles to the semiconductors has been found to play a dominant role in modulating semiconductor reactivity. Hot electron injection from plasmonic particles to semiconductors has been demonstrated, and hybrid plasmonic-semiconductor materials have been utilized in a growing number of investigations of photovoltaic devices (Atwater and Polman, 2010; Linic et al., 2011; Thomann et al., 2011; Mubeen et al., 2013; Clavero, 2014) and photocatalysts (Zhao et al., 1996; Christopher et al., 2011; Marimuthu et al., 2013; Mukherjee et al., 2013). Combining plasmonic nanoparticles with a semiconductor photocatalyst can significantly boost the efficiency of light absorption as well as induce high local electric fields for charge separation. More importantly, the semiconductor can serve as a sink for hot electrons generated in the SPR process, extending their lifetimes to be in the nanosecond time scale (Zhu, 1994). Many of these investigations have shown enhancements of the photocatalytic rates at their surfaces. As illustrated in Figure 3B, the concept is that the excitation of the localized SPR of nanoparticles will generate hot electrons and hot holes that can be injected into the conduction band or the valence band, respectively, of the metal oxide. Thus, the oxidation and reduction reactions can occur separately over the plasmonic nanoparticle and metal oxide. In addition, the operational wavelength range of such systems can be tuned by controlling the SPR resonance frequency from the infrared to ultraviolet photon energies.

However, the efficient utilization of the hot electrons has been a formidable challenge because of the extremely short relaxation lifetimes, on the order of a few picoseconds, owing to the multiple fast relaxation processes including electron-electron scattering, electron-interface scattering, electron–phonon interaction, photoluminescence, etc. (Linic et al., 2011; Manjavacas et al., 2014; Ma, X. C. et al., 2016). When combining the plasmonic particles with conventional semiconductors, the hot electron injection efficiency (HEIE) is usually low. The Schottky barrier (i.e., the energy barrier across the metal and the semiconductor interface, described later) is the main hurdle for the low efficiency. Practical factors, e.g., the occupation of surface oxygen vacancies of titania oxide by Au atoms during the synthetic process, may increase the barrier and expand the space charge region, which significantly decreases the HEIE (Ma et al., 2014b). Strategies to most efficiently use the hot charge carriers generated in an SPR process are currently under intensive investigation (Ma, X. C. et al., 2016). Current efforts to improve the hot electron injection efficiency include designing composite structures so that the hot electrons have sufficient momentum to cross the interface (Giugni et al., 2013), chemically modifying the surfaces so that efficient electronic coupling between the plasmonic particle and the semiconductor occurs (Tamura et al., 2009), and applying pressure, which shifts the positions of the valence/conduction bands and favors lowering the barrier height (Ma, X. et al., 2016; Lv et al., 2018). However, these efforts have so far not yielded an adequately efficient design to capture and harness the hot electrons.

### Ferroelectric Surfaces for Extracting Hot Carriers of Plasmonic Nanoparticles

**Figure 4:**
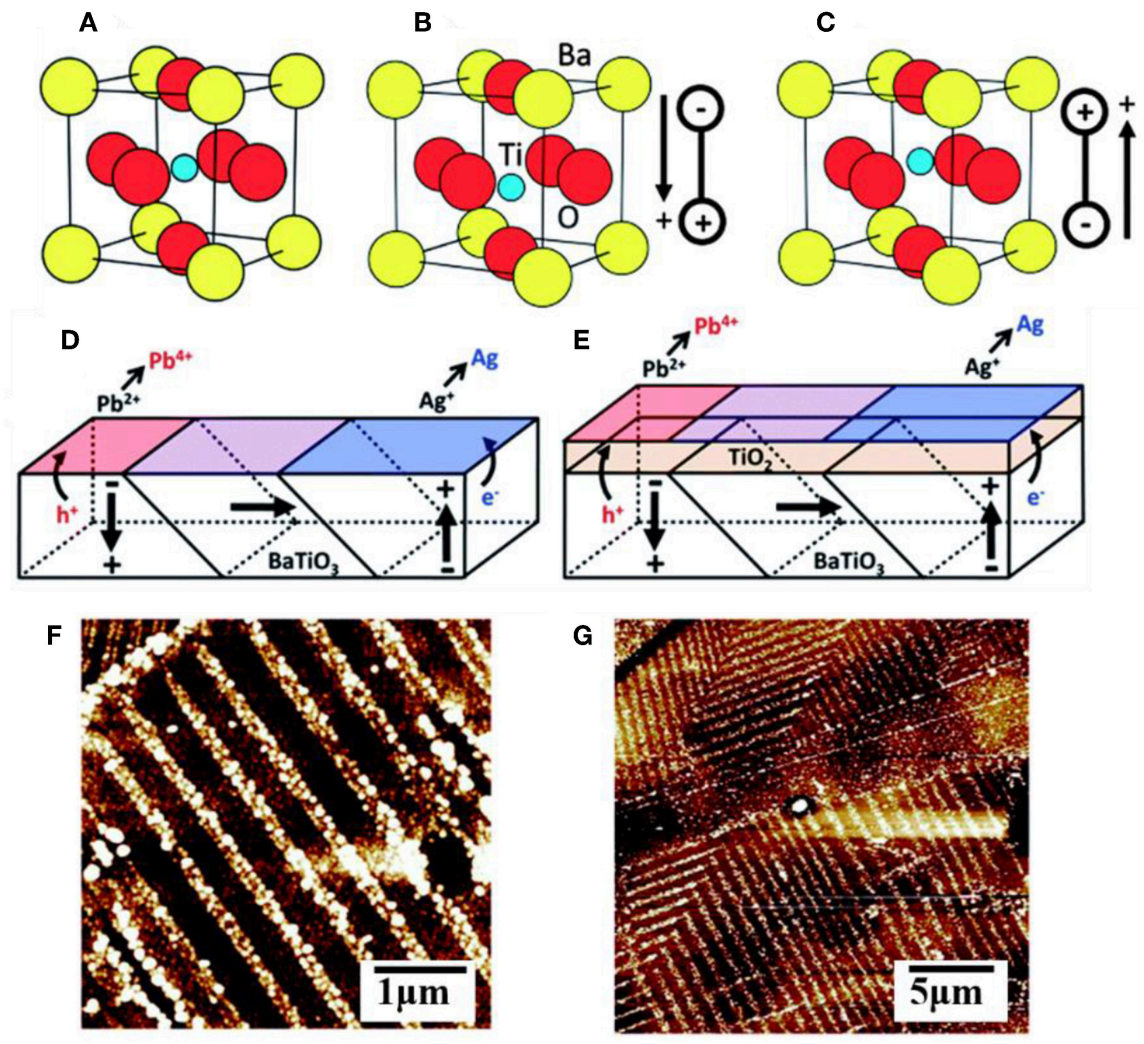
The unit cell of BaTiO_3_ in the **(A)** cubic form and **(B,C)** in its ferroelectric polymorph showing two different polar orientations. A multidomain surface with multiple polar orientations without **(D)** and with **(E)** a TiO_2_ layer coating. AFM images of a (100) oriented BaTiO_3_ surface after photochemical deposition of metallic Ag (white contrast) without **(F)** and with **(G)** the TiO_2_ layer coating. Adapted with permission from Giocondi and Rohrer (2001). Copyright 2001 American Chemical Society.

Ferroelectric materials have been extensively used in microelectronics, memory storage devices, temperature sensors and switches, thin film capacitors, and more (Daniel and Astruc, 2004). They are characterized as possessing a large, reversible electric polarization within their crystalline grains owing to a displacement of the centroid of positive and negative charges, as illustrated in Figures 4A–C for BaTiO_3_ in the symmetric cubic (*Pm-3m*) or distorted tetragonal (*P4mm*) polymorphs of the perovskite structure type. Many ferroelectrics with the common ABO_3_ composition crystallize in perovskite-type structures that exhibit polar distortions. For example, ferroelectric PbTiO_3_ exhibits a tetragonal structural distortion below a Curie temperature of ~495°C and with a large, spontaneous ferroelectric polarization of ~80 μC cm^−2^ that persists even in the absence of an applied electric field (Qin et al., 2007; Arney et al., 2011; Cao et al., 2012b; Kakekhani and Ismail-Beigi, 2016; Kakekhani et al., 2016; Wang et al., 2016). A freshly prepared sample will have a large number of individual grains, or domains, with random orientations that are separated by domain walls, shown in Figures 4D,E for BaTiO_3_. Unit cells within the domain have a shared polarization direction, labeled with arrows, owing to the collective displacements of the cations in the same directions. However, changes to the displacement of the cations around the oxygen anions can be induced by application of an external electric field, causing the polarization of individual grains to eventually be aligned with the external field in a process known as poling. This process is reversible, and thus ferroelectrics have a switchable polarization (Kalinin and Bonnell, 2001; Bonnell and Kalinin, 2002; Streiffer et al., 2002; Daniel and Astruc, 2004; Cai et al., 2007).

Many recent studies have suggested that the surfaces of a ferroelectric (FE) can yield potentially optimal configurations for achieving significant efficiency enhancements of charge separation and charge injection across interfaces. The spontaneous polarization can facilitate electron-hole separation within the ferroelectrics through coulombic interactions (Yun and Altman, 2007), and also where the domain walls can act as charge trapping centers or as reactive surface sites (Qin et al., 2007; Sakar et al., 2016; Zhu et al., 2018). Many prior studies have demonstrated that the internal polarization of a ferroelectric can result in the spatial segregation of oxidation and reduction reactions over its oppositely polarized surfaces. For example, photochemical deposition studies on BaTiO_3_, BiFeO_3_, and other ferroelectrics have shown spatial separation of the light-driven oxidation of Pb^2+^(aq) to PbO_2_(s) and the light-driven reduction of Ag^+^(aq) to Ag(s) (Li et al., 2014). An illustrative case is depicted in Figure 4 for BaTiO_3_, wherein atomic force microscopy shows that its positively polarized surfaces are preferentially the sites for reductive reactivity and its negatively polarized surfaces are the preferential sites for oxidative reactivity. Recent calculations have shown that positively and negatively polarized domains, which are separated by 180°, lead to the highly efficient separation of the charged carriers to the oppositely polarized surfaces with internal quantum efficiencies that can exceed 90% (Glickstein et al., 2016). The advantage is that both types of carriers, i.e., majority and minority carriers, can be separated and reacted over adjacent surfaces without the need for extended diffusion pathways through a film, external circuit, or via redox shuttles in solution. Efficient charge separation within ferroelectrics is thus an attractive feature for photocatalytic reactions such as water splitting. For example, using the ferroelectric PbTiO_3_, Maggard et al. investigated its photocatalytic activity for the reduction and oxidation of water and showed that high activities for both reactions occur for nanoparticles as well as micron-sized particles (Arney et al., 2011). A limiting feature of ferroelectrics is that, with a few rare exceptions, they usually have large bandgap sizes that fall within the ultraviolet energy range. For example, PbTiO_3_ has a bandgap that falls at the edge of the visible range at ~2.7 eV and does not absorb light with a wavelength longer than ~460 nm. Thus, the many recent investigations into ferroelectrics have focused on their capabilities to increase charge separation or charge extraction when combined with semiconductors with smaller bandgap sizes (Bowen et al., 2014).

Recent results, described below, have suggested that the tethering of plasmonic nanoparticles to the surfaces of a ferroelectric can lead to an optimal configuration for achieving an enhanced extraction of hot carriers from them. The basic idea is that a ferroelectric with a large surface polarization can facilitate the fast charge extraction of hot carriers from plasmonic nanoparticles. The unique advantages of this combined system include the capability to independently manipulate the electric polarization of the ferroelectrics as well as the local SPR wavelengths of the nanoparticles in order to achieve synergistic energetic conditions at their interface to optimize the injection of the hot charge carriers. In addition, better charge separation can be achieved in this combined system in which the domain walls of the ferroelectrics can serve as the charge trapping centers or as reactive surface sites (Qin et al., 2007; Sakar et al., 2016; Zhu et al., 2018). Furthermore, all potential advantages of an interfaced metal-semiconductor system are inherited, e.g., enhanced optical excitation of the semiconductor due to the local field effect (Ma et al., 2014b). The underlying mechanism for the enhanced charge injection involves a tuning of the band bending and the Schottky barrier height at the semiconductor-metal interface, which has also represented a long-standing problem in the physics and chemistry of electronic materials. The impacts of the ferroelectric polarization and the composition of the metallic particles are described below, together with recent experimental results that have demonstrated that the hot charge carriers can be injected into ferroelectrics (Atwater and Polman, 2010; Grinberg et al., 2013).

## Models of Metal-Ferroelectric Interfaces and the Schottky Barrier

Currently very little is known, either theoretically or experimentally, about the hot charge injection from plasmonic particles to ferroelectric materials from a perspective of light-driven catalysis, e.g., such as for water splitting reactions. Investigations of the interfaces between metals and ferroelectrics have focused on their potential applications in solid-state photovoltaic devices, wherein the ferroelectric both functions as the light-absorbing component as well as the underlying driving force for charge separation. A model of the electronic structure and band bending at semiconductor surfaces interfaced with a metal is described first, followed by the impact of the ferroelectric polarization at this interface. This is critical to understanding the experimental photocurrents and photocatalytic properties in recent investigations.

### Schottky Barrier Height at Semiconductor-Metal Interfaces

At the surface of a crystalline semiconductor, the periodic pattern of chemical bonds is interrupted and leads to the formation of dangling bonds from the incomplete atomic coordination environments. These dangling bonds can be responsible for the formation of localized surface states with energies that fall within the band gap of the semiconductor. An inversion layer, or depletion region, is formed near the surfaces, which results from a flow of its majority carriers, i.e., either *n*-type or *p*-type, either toward or away from the surfaces to fill the surface states and reach electrochemical equilibrium (Sze and Ng, 2006). The interface states are filled up to the charge neutrality level (CNL), above which these states are empty (Tersoff, 1986; Robertson and Chen, 1999). The charge transfer between the surface states and the bulk of the semiconductor causes the bands to bend at the interface (McCaldin et al., 1976; Spicer et al., 1980).

**Figure 5:**
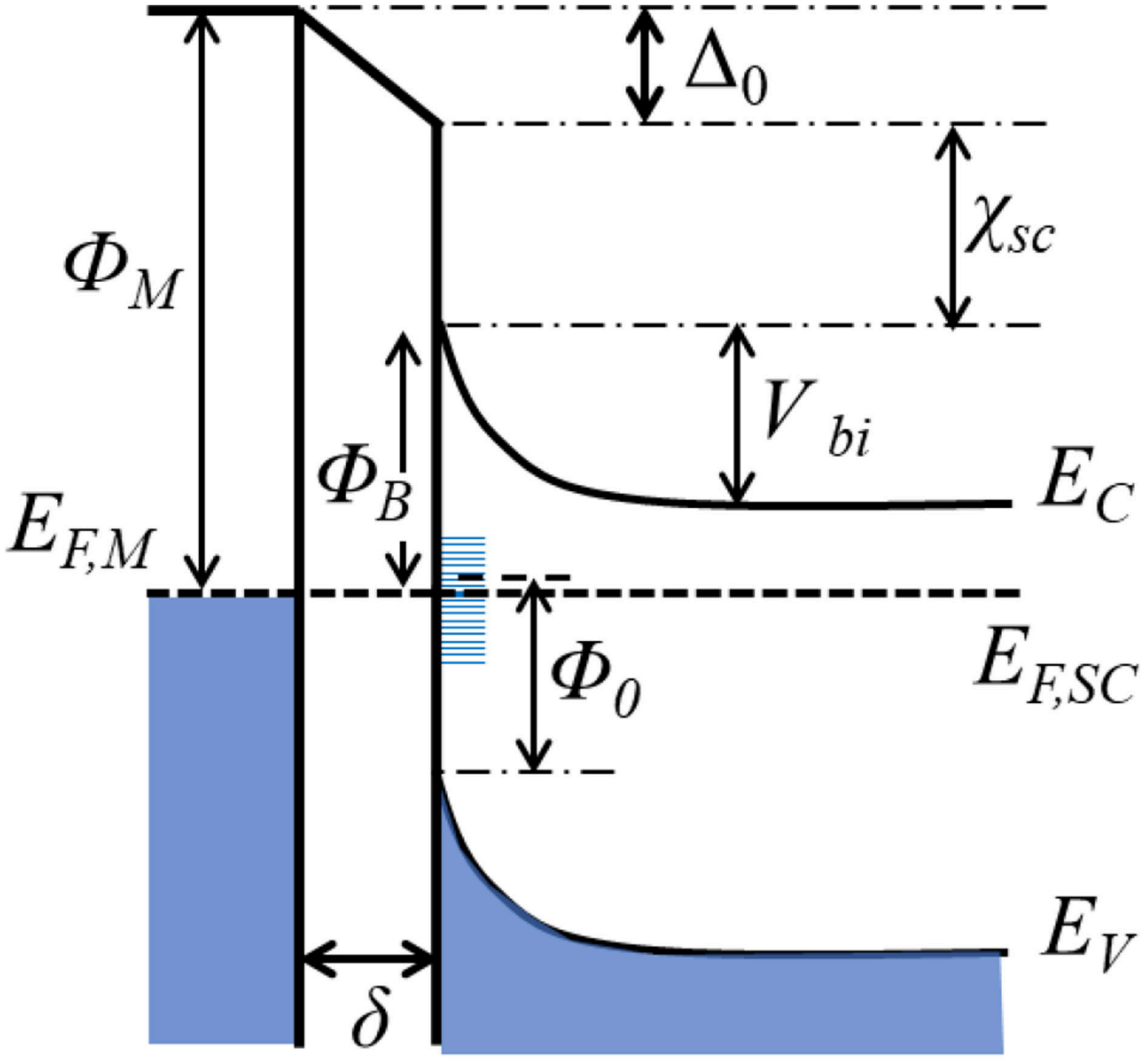
Energy diagram of a metal-semiconductor(*n*-type) contact with an interfacial layer. *Φ*_*M*_, metal work function; *E*_*F,M*_ and *E*_*F,SC*_, Fermi levels of the metal and semiconductor. χ_SC_: semiconductor electron affinity. *E*_*C*_*, E*_*V*_: Energy levels at the edges of the conduction and valence bands. *Φ*_*B*_: Schottky barrier height. V_bi_: built-in potential. δ: interfacial thickness. Δ_0_: voltage drop across the interface. *Φ*_0_: energy change between the valence band edge and charge neutrality level.

When a semiconductor is in contact with a metal, the difference in their work functions causes electrons to transfer between them. For example, if the metal work function is larger than that of the semiconductor, the electrons will flow from the semiconductor to the metal until the Fermi levels have equilibrated. Electron transfer from semiconductor to the metal (for *Φ*_*M*_ > *Φ*_*SC*_) or from metal to semiconductor (for *Φ*_*M*_ < *Φ*_*SC*_) will cause band bending upward or downward, respectively, at their interfacial region (Zhang and Yates, 2012). For example, Figure 5 shows an example whereby *Φ*_*M*_ is larger than *Φ*_*SC*_ and upward band-bending has resulted. This junction, known as a Schottky junction, represents a potential energy barrier that manifests as a rectifying electrical contact between the metal and the semiconductor, i.e., the flow of electrons in this case occurs most easily from the semiconductor to the metal. The energy barrier for electrons to flow in the reverse direction from the metal and into the conduction band (CB) of the semiconductor is known as the Schottky barrier (*Φ*_*B*_). The flow or injection of electrons across this barrier depends in an exponential manner on the height of the Schottky barrier and is thus one of the most critical properties of a metal-semiconductor interface. In the simplest case, the energetic height of the Schottky barrier follows the Schottky-Mott relationship, which for an *n*-type semiconductor is given by Equation (1):

(1) $$\begin{array}{l} \Phi_{B}=\;\Phi_{M}-\;\chi_{\text{SC}} \end{array}$$

where χ_SC_ is semiconductor electron affinity. It can be taken that the voltage drop arising from the formation of an interface dipole, i.e., Δ_0_, is considered to be zero for this simplified case. The band bending at the surface of the semiconductor functions as the driving force for charge separation, and is given by the built-in potential (*V*_*bi*_) in Equation (2):

(2) $$\begin{array}{l} V_{bi}=\Phi_{B}\;-\left(E_{C}-E_{F,SC}\right) \end{array}$$

where *E*_*F,SC*_ is the Fermi level of the semiconductor, and *E*_*C*_ is the conduction band edge of the semiconductor. Hot electron injection will be favored when *Φ*_*SC*_ < *Φ*_*M*_ and the semiconductor bands bend up, while hot hole injection will be favored when *Φ*_*SC*_ > *Φ*_*M*_ and the bands bend down. Thus, in both cases, the unidirectional injection of hot carriers across the interface leads to their efficient separation via a drift current in the semiconductor that flows away from the metallic nanoparticle. Note that in this review article, the focus is exclusively on the injection of hot electrons, which are relatively better characterized and typically have high mobility. For a *p*-type semiconductor, the bands typically bend downward due to its low-lying Fermi level. In this case, the potential barrier (*V*_*bi*_) will be nil and instead an accumulation layer will form. Thus, the interfaces between plasmonic metals and *p*-type semiconductors will not be favorable for hot electron injection. Hence, only *n*-type semiconductors are covered in this review.

With rare exceptions, however, the Schottky barrier height has been found to show a relatively weak correlation with the metal work function as predicted by Equation 1 (Tung, 2000; Dimoulas et al., 2006). The cause of this is known as Fermi level pinning and is driven by a large concentration of mid-gap states that form at the metal-semiconductor interface arising from metal-induced gap states, disorder-induced gap states, defect states, and other surface defects. These states become filled by charge flow from the semiconductor and result in an interface dipole and a voltage drop across the interface. Different models have been proposed to account for this phenomenon, including the most popular Cowley-Sze model, which takes semiconductor surface states and Fermi level pinning into consideration. The revised form of the Schottky barrier height is defined by the following Equation (3) (Bardeen, 1947; Cowley and Sze, 1965):

(3) $$\begin{array}{l} \Phi_{B}=S\left(\Phi_{M}\;-\;\chi_{\text{SC}}\right)+\left(1-S\right)\left(E_{g}-\Phi_{0}\right) \end{array}$$

Here, *E*_*g*_ represents the band gap of the semiconductor; *Φ*_0_ is the energy difference between the CNL and the valence band edge of the semiconductor at the surface, and *S* is the pinning parameter depending on the surface state density of the semiconductor. Practically, *S* has been found to fall between 0 and 1, where “0” stands for maximum and “1” stands for minimum pinning, respectively (Cohen, 1979). Monch empirically found that the pinning parameter, *S*, follows a linear approximation (Mönch, 1987):

(4) $$\begin{array}{l} S=1/\left(1+0.1{\left(\varepsilon -1\right)}^{2}\right) \end{array}$$

where the dielectric constant ε is taken as the square of the refractive index of the semiconductor. For ionic semiconductors such as ZnO or ZnSe, *S* approaches 1 (Schottky limit) and the Cowley–Sze model reduces to Schottky–Mott model (Equation 1). For covalent semiconductors, *S* approaches 0 (Bardeen limit). When the interface state density approaches infinity, then the Cowley-Sze model takes the form of Equation (5):

(5) $$\begin{array}{l} \Phi_{B}=\;E_{g}-\Phi_{0} \end{array}$$

In this case, the Fermi level of the metal is pinned to CNL, and hence the Schottky barrier height is independent of the metal work function (Bardeen, 1947; Kurtin et al., 1969). This relationship was found by Dimoulas et al. to be consistently followed at metal/Ge interfaces (Dimoulas et al., 2006). However, the Schottky barrier height is known to be intricately dependent upon the specific interfacial bonding, i.e., sensitive to surface orientation, surface relaxation, and other factors (Tung, 2014). It has been reported in many cases to typically vary by amounts of ± 0.2 to 0.8 eV for even the same metal and semiconductor combinations. Thus, the formation and height of the Schottky barrier must be investigated for each specific metal-semiconductor combination and under identical conditions of their preparation.

### Ferroelectric Control Over the Schottky Barrier Height

**Figure 6:**
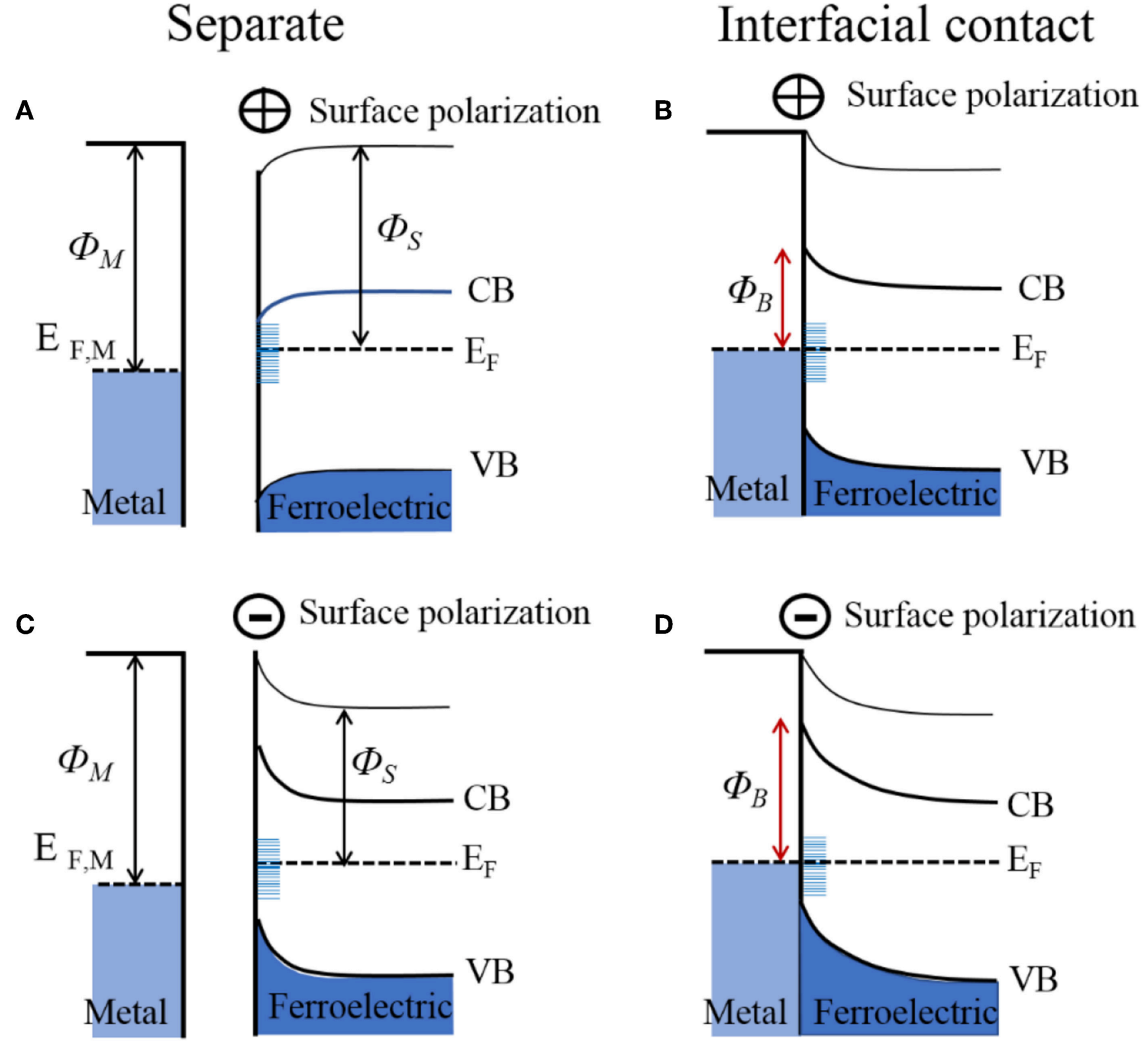
Electronic energy levels of an *n*-type ferroelectric for its surfaces that are positively charged (upper; **A,B**) and negatively charged (lower; **C,D**), shown before and after interfacial contact with a metal. *Φ*_*M*_, metal work function; *Φ*_*S*_, semiconductor work function; *Φ*_*B*_, Schottky barrier height; *E*_*F*_, Fermi level; CB, conduction band; VB, valence band.

At metal-ferroelectric interfaces, the spontaneous surface polarization must also be accounted for when considering the band bending and Schottky barrier height. This is a consequence of the fact that the ferroelectric polarization at the surface will establish an additional interface potential and, depending on its orientation, will modify the surface binding energies and Schottky barier height with the metal. The effect of the surface polarization has been observed in the photocatalytic reactivity of multi-domain ferroelectrics, notably including PbTiO_3_, Pb(Ti_1−x_Zr_*x*_)O_3_ (PZT), BaTiO_3_, and BaBiFeO_3_ (Inoue et al., 1989; Apostol et al., 2013). In these cases, the different orientations of the surface polarization, e.g., represented as P(+), P(–), or P(0), can cause light-driven reduction reactions at P(+) surfaces and oxidation reactions at P(–) surfaces, as shown in Figures 4F,G for BaTiO_3_ (Schultz et al., 2011). When P(–) and P(+) surface polarizations occur at the surface of the ferroelectric, the interface dipole leads to a negative and positive impact on the band bending, respectively, illustrated in Figures 6A,C. Thus, with bandgap excitation of electron-hole pairs in the depletion region, the electrons are separated and driven to P(+) surfaces and the holes to the P(–) surfaces. For a ferroelectric in contact with a metal, the difference in the work function of the metal and semiconductor will equilibrate with the flow of charged carriers between them, and either strengthen or weaken the surface ferroelectric polarization, thus modifying the Schottky barrier height. Shown in Figures 6B,D is the example of a metal in interfacial contact with the P(+) and P(–) of a ferroelectric, e.g., Au in contact with PZT. Pintilie and Alexe quantitatively expressed the band-bending of ferroelectric semiconductors by introducing the surface polarization as a sheet of surface charge located at a finite distance from the electrode interface, with a modified built-in potential *V*_*bi*_′ at the ferroelectric surface given by Equation (6) (Jones et al., 2008):

(6) $$V_{\text{bi}}{}^{\prime }=\;V_{bi}\;\pm \;P\delta /\varepsilon_{0}\varepsilon_{\text{st}}$$

where *P* is the component of the polarization that is perpendicular to the sample surface, ε_0_ is the permittivity of free space, ε_*st*_ is the low-frequency (or static) dielectric constant, and δ is the interface thickness layer which is typically assumed to be on the order of a single unit cell. This gives a quantitative estimate of the degree of band-bending at polarized ferroelectric surfaces, as has been confirmed by recent investigations on PZT, BaTiO_3_, and BiFeO_3_ (Pintilie and Alexe, 2005; Pintilie et al., 2005, 2014). For *n*-type ferroelectric semiconductors interfaced to metals, the P(–) polarized surface will cause the Schottky barrier to be higher as compared to the unpolarized P(0), shown in Figure 6D. Correspondingly, the P(+) polarized surfaces will serve to either lower or completely reverse the Schottky barrier, shown in Figure 6B. Whether the bands on the P(+) surface bend downward or upward depends on the magnitude of the band-bending introduced by the spontaneous polarization, as determined by Equation (6). However, the surface polarization of many strong ferroelectrics can have a predominating impact, e.g., changes to the Schottky barrier heights of up to ~1.1 eV for BaTiO_3_ and up to ~0.9 eV for PZT (Pintilie et al., 2014).

## Experimental Investigations of Schottky Barrier Heights and Band Bending at Ferroelectric-Metal Interface

### Manipulation of Interfacial Electrical Transport and Charge Separation

**Figure 7:**
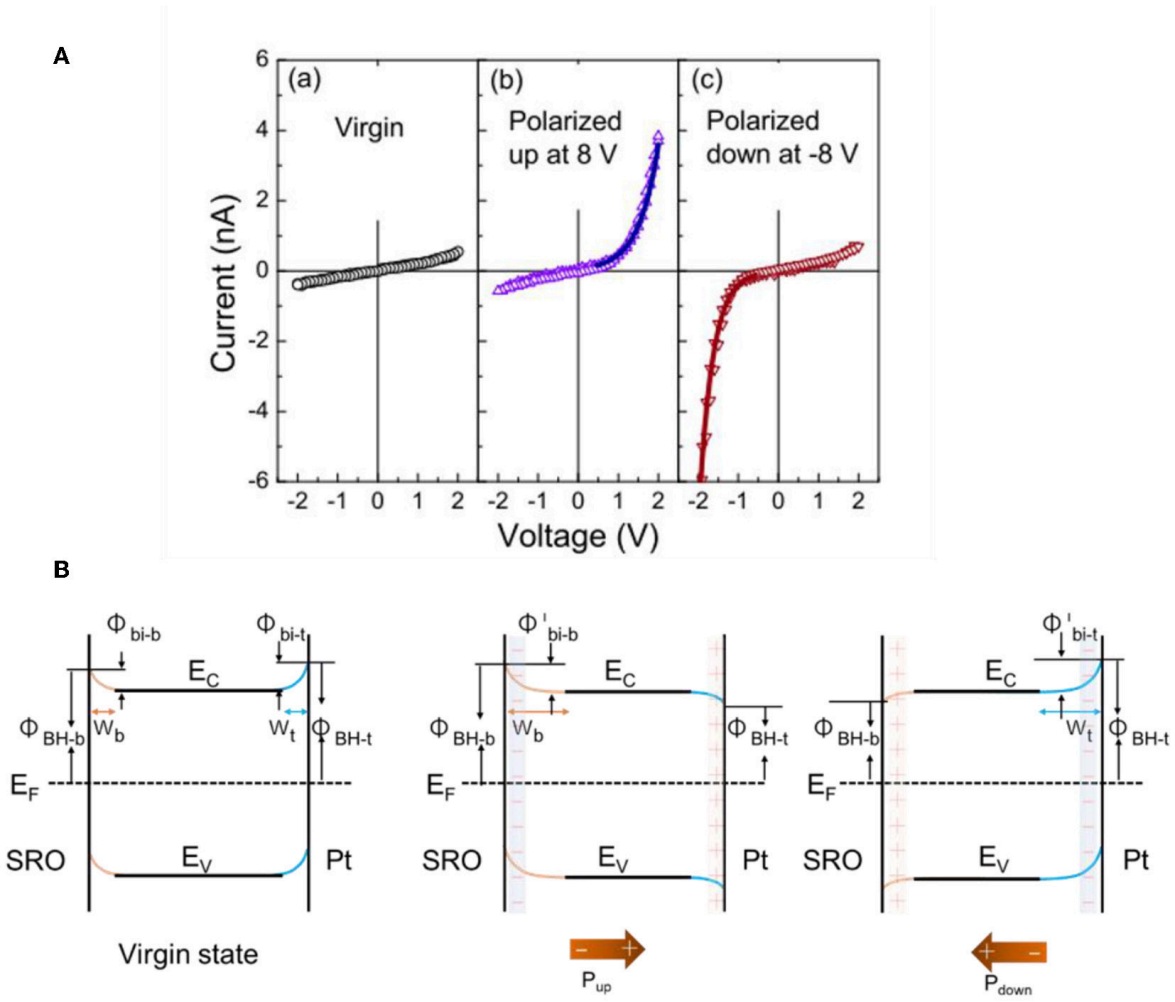
Band-bending at SRO/BFO/Pt heterojunctions. **(A)** I–V characteristics at left: virgin state, middle: polarized up state, and right: polarized down state. **(B)** Proposed energy band diagrams at different ferroelectric polarization states. Left: virgin state. Middle: polarization up state. Right: polarization down state. Figures adapted from Wang et al. (2011). Copyright 2011 AIP Publishing.

The strong polarization-dependent manipulation of the Schottky barrier at ferroelectric-metal interfaces has been investigated in several recent studies, such as has been typically characterized by current-voltage (I–V) curves or X-ray photoelectron spectroscopy (Yang et al., 2010; Alexe and Hesse, 2011; Cao et al., 2012a). For example, recent studies have focused on their rectifying electrical behavior, such as to yield switchable diodes, or in facilitating charge separation and transport in solar cells with the ferroelectric as the light absorber. It has been shown that by reversing the polarization of the ferroelectric the Schottky barrier can be reversed and induce a switchable diode behavior at the interface (Wang et al., 2011). For example, Wang et al. characterized the interfacial Schottky barrier between platinum/BiFeO_3_ (BFO) using current-voltage hysteresis, shown in Figure 7A. A thin film of ferroelectric BFO was grown over a SrTiO_3_ (001) (SRO) single crystal and ~100 μm platinum dots were deposited over the BFO film. The Pt and SRO acted as the two electrodes to measure the electrical current and exhibited distinct hysteresis behavior that indicated large resistive switching between −6 and +6 V. Both the positive and negative polarization were characterized independently. First, the SRO/BFO/Pt heterojunction was polarized to induce upward and downward polarization, shown in Figure 7B with the two polarization directions. The I-V curves show that for the virgin state without poling, the current is very small and increases linearly with voltage at both positive and negative bias. For the polarized-up state, the current increases exponentially with the positive applied voltage, i.e., SRO as the anode and Pt as the cathode, but increases much more slowly with negative applied voltage. This shows a forward diode-like behavior. For the polarized down state, the current shows a reverse diode behavior. This switchable diode behavior demonstrated that the Schottky barriers between the *n*-type semiconductor ferroelectric BFO and metallic electrodes have been changed by the polarization switching. Changes in the interfacial band structure with the two different polarizations for the SRO/BFO/Pt sandwich are illustrated in Figure 7B. In the virgin state, the Schottky barrier heights at both interfaces are sufficiently large owing to the larger work functions of the SRO and Pt contacts compared to that for BFO. This is consistent with the very small currents. For positive polarization, the Schottky barrier becomes reversed on the Pt side, forming an Ohmic contact, with the cell behaving like a forward diode as shown in Figure 7B (middle). For the opposite ferroelectric polarization, the Schottky barrier becomes reversed to form an Ohmic contact on the SRO side, and the cell behaves like a reverse diode [Figure 7B (right)]. These observations are consistent with Equation 6 and demonstrate the modulation of the Schottky barrier using the ferroelectric polarization at the interfaces.

**Figure 8:**
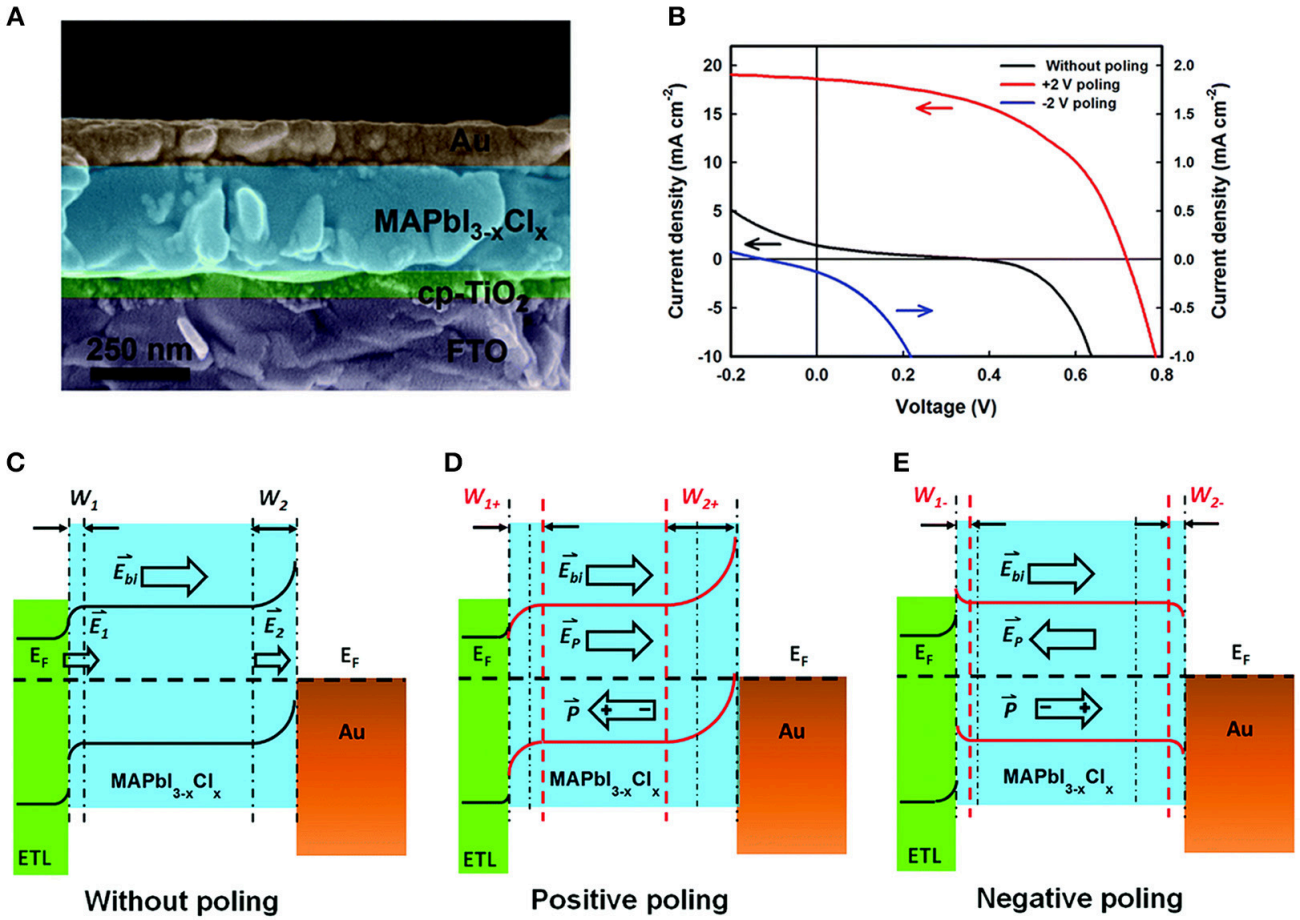
Band-bending at TiO_2_/MAPbI_3−x_Cl_x_/Au heterojunctions. **(A)** SEM of the ferroelectrics-based solar cell (left) and J–V plot (right). **(B)** Proposed electronic band structure under different polarization conditions. **(C)** Without poling. **(D)** Positive poling. **(E)** Negative poling. Figures adapted from Chen et al. (2015). Copyright 2015 Royal Society of Chemistry.

Ferroelectric-based solar cells have been the subject of many recent investigations owing to their switchable photocurrents and higher-than-bandgap open circuit photovoltages, including for BiFeO_3_, PZT, BaTiO_3_, and Bi_2_FeCrO_6_ (Moubah et al., 2012; Zenkevich et al., 2014; Zheng et al., 2014; Nechache et al., 2015). For example, modification of the Schottky barrier height and depletion region at a ferroelectric-metal interface were investigated by Chen et al. and found to achieve solar cells with power conversion efficiencies of up to 6.7% and photocurrents of ~19 mA cm^−2^ on electrically-poled samples (Chen et al., 2015). The ferroelectric methylammonium lead trihalide (MAPbX_3_) (FE-MAP) was used with a configuration of TiO_2_/FE-MAP/Au, shown in Figure 8A. Electric poling of ±5 V DC voltage was used to switch the domain alignments (Figure 8B). The proposed band structure of all three subunits of the solar cell is illustrated in Figures 8C–E. In the pristine state, i.e., no poling, a Schottky barrier forms at the ferroelectric/Au interface owing to the greater work function of Au. At the other side, a compact TiO_2_ thin layer serves as the *n*-type electron transport layer (ETL). The bands in MAPbX_3_ are bent downward and upward at the TiO_2_/ferroelectric and the ferroelectric/Au interfaces, respectively, shown in Figure 8C. Upon irradiation of the cell the two depletion regions drive the separation of the electron-hole pairs, with larger depletion regions favoring a more efficient charge separation. With no electrical poling, the solar cell gives a power conversion efficiency of only ~0.09%, a photocurrent of 1.47 mA cm^−2^ and an open circuit photovoltage of 0.37 V. However, a positive electric poling of the ferroelectric at +0.2 V leads to significantly enhanced band bending, i.e., a higher Schottky barrier height at the ferroelectric-Au interface that widens the depletion region for more efficient charge separation, illustrated in Figure 8D. This gives a dramatically higher power conversion efficiency of 6.7%, a photocurrent of 18.5 mA cm^−2^ and an open circuit voltage of 0.72 V. Thus, the direction and magnitude of the surface polarization can be effectively used to manipulate the interfacial barrier heights, which for solar cell designs can yield a more efficient charge separation and higher open circuit voltage.

### Facilitating Hot Carrier Extraction Using Ferroelectrics

While there are many recent investigations into the utility of ferroelectric-metal interfaces for charge separation in solar cells, currently very few studies have focused on the effects of the photo-excitation of the localized SPR of the metallic nanoparticles. The excitation of the localized SPR can lead to the efficient absorption of photons and the generation of hot carriers with sufficiently high energies compared to the Schottky barrier in order to facilitate injection. For example, Schottky barrier heights for several metal-ferroelectric combinations have been measured to fall within the range of ~0.1 to 0.9 eV, such as those found for PZT-Au interfaces of ~0.3 eV and BaTiO_3_-Cu interfaces at 0.2 eV (Pintilie et al., 2014). Thus, the excitation of the localized SPR and the generation of hot carriers at energies only a few hundred millielectron volts above the Fermi level are sufficient for injection over the Schottky barrier and into the ferroelectric in many cases. The critical role of the barrier height in the injection of electrons from metals into semiconductors was first modeled by Fowler in the early 1900s, as given in Equation (7) (Fowler, 1931):

(7) $$\begin{array}{l} Y_{Fow}\left(h\nu \right)\approx \frac{1}{8E_{F}}\frac{{\left(h\nu -\phi_{b}\right)}^{2}}{h\nu } \end{array}$$

where *Y*_*Fow*_ is the Fowler yield, *h* is Planck's constant; ν is the incident light frequency; ϕ_*B*_ is the barrier height at the interface, and *E*_*F*_ is the Fermi energy of the metal. This is a semiclassical model with the basic assumption that the kinetic energy of the hot electrons normal to the barrier must be greater than the barrier height. This equation shows that injection of hot carriers is proportional to the square of the barrier height and can be manipulated by interfacial ferroelectric polarization. Theoretical calculations have been used to determine the optimal energetic structure at the interface and have suggested efficiency limits of about 1–10% under the most optimistic conditions (Leenheer et al., 2014). Further studies, however, which take into account anisotropic particle shapes that impact the electron-phonon scattering events and diffuse reflections, have shown that these factors can significantly increase the hot electron injection efficiency up to a higher efficiency limit of about 20% (Blandre et al., 2018), which is a factor of four greater than Fowler's limiting efficiency. Only a few recent investigations have reported the use of metal-ferroelectric interfaces to further enhance the hot carrier injection efficiency, as described below.

#### Hot Charge Injection at Buried Nano-Au/PZT Interfaces

The earliest research efforts focused on extracting hot charge carriers using non-ferroelectric semiconducting oxides, especially TiO_2_, as described above. Despite the intense surge of interest in this area, the resulting efficiencies are usually very low, and a detailed understanding of the hot carrier process has remained unclear. Ferroelectric-interfaced plasmonic nanoparticles represent a relatively unexplored and fertile research ground, wherein by contrast, the ferroelectric polarization can be used to systematically manipulate the Schottky barrier height and the extent of the depletion region as a function of the ferroelectric surface polarization and applied poling field. In the first reported study by Wang et al., solar cells were constructed using a ferroelectric Pb(Ti_1−x_Zr_*x*_)O_3_ (PZT) film that was patterned with an array of Au nanoparticles with lateral dimensions of 270 × 270 nm^2^ and a thickness of ~60 nm. For this relatively large size of Au nanoparticles, the radiative re-emission of photons significantly predominates compared to hot carrier generation, as described in the Background section. Further, the distribution of hot carriers is calculated to be only on the order of a few tenths of an eV different than the Fermi energy. However, prior studies have found the Schottky barrier of PZT-Au interfaces to be as low as ~0.1 eV (Pintilie et al., 2014). The photocurrent was still boosted by nearly an order of magnitude when aligning the polarization direction in PZT and reversing the Schottky barrier with the electrolyte. This increased the external quantum efficiency from ~0.1 to 0.3% at 600 nm, which is at significantly lower energies than the PZT band gap of ~3.6 eV.

**Figure 9:**
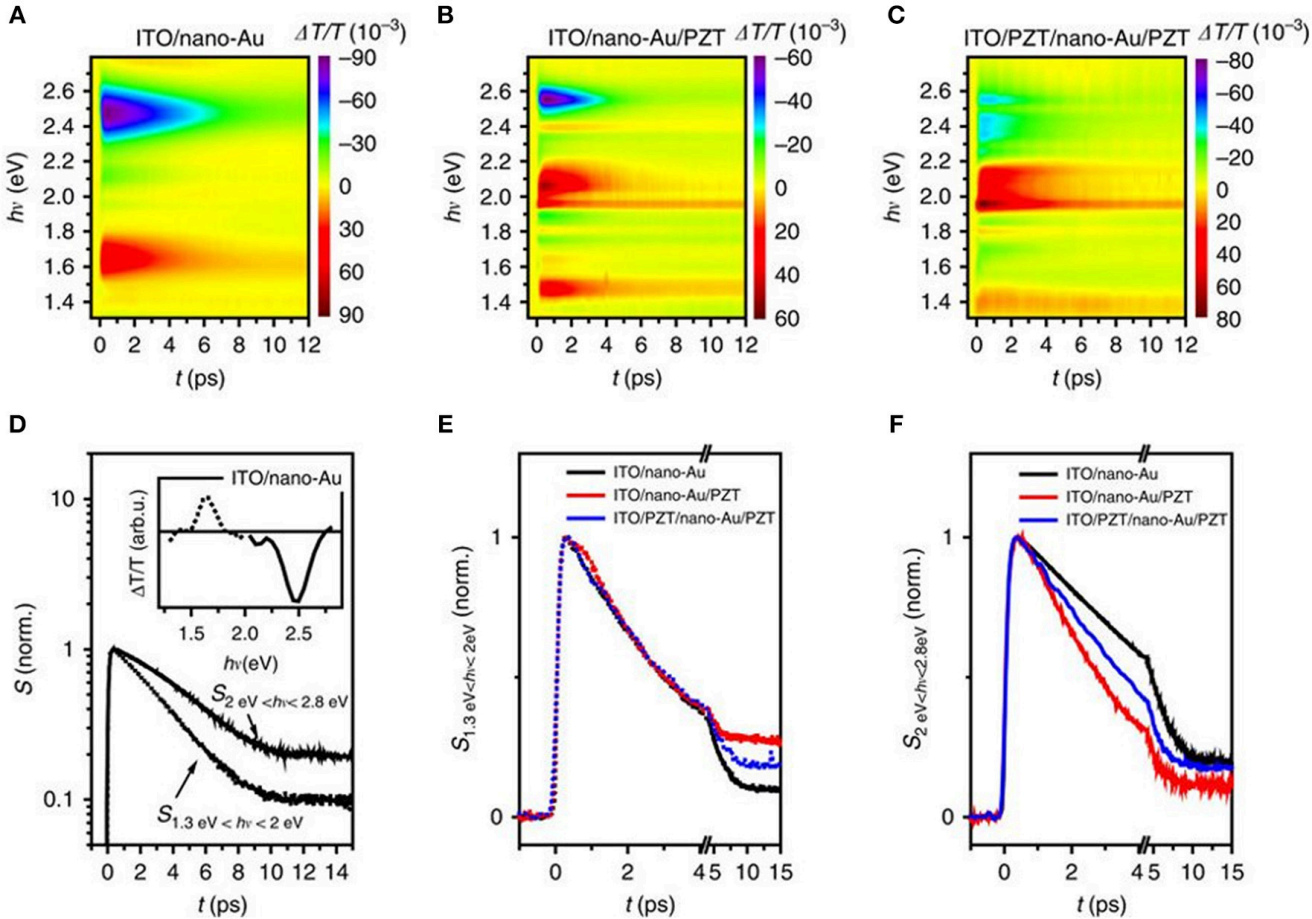
Transient absorbance spectroscopy of the plasmonic-ferroelectric hybrids. Time evolution of ΔT/T for **(A)** ITO/nano-Au, **(B)** ITO/nano-Au/PZT, and **(C)** ITO/PZT/nano-Au/PZT, following photoexcitation with 70 fs ultraviolet pulses at 400 nm. **(D)** The time evolution of the response for ITO/nano-Au in the low and high frequencies, respectively. Comparison of the **(E)** low-frequency and **(F)** high-frequency dynamics between the three nano-Au hybrids. Figure adapted from Wang et al. (2016). Copyright 2016 Nature Publishing Group.

Broadband transient absorbance (TA) measurements were used to characterize the hot charge transfer between the Au nanoparticles and PZT. Femtosecond optical pulses centered at a wavelength of 400 nm were used for excitation, i.e., at photon energies below the PZT band gap. The nano-Au array on ITO/glass showed two distinct, spectrally well-separated components in the transient spectra, shown in Figures 9A,D: one increased transmission peak (ΔT/T, or decreased absorption peak) at 1.65 eV and the other reduced transmission peak at 2.5 eV. The former was linked to the photoinduced changes in the localized SPR absorption at 1.5 eV, and the latter was attributed to the bulk-like response of Au, governed by the photoinduced changes in the density of states for the d-band to Fermi level transition. A reduced transmission indicates the increased density of states after photo-excitation.

For devices of ITO/nano-Au/PZT and ITO/PZT/nano-Au/PZT, shown in Figures 9B,C, the time evolution of the low energy (1.3–2 eV) and the high energy (2.0–2.8 eV) responses were analyzed (Figures 9E,F). The relaxation dynamics of the low-energy part are similar for all three devices and can all be fitted with a single exponential decay. However, the high energy component shows a non-exponential relaxation, with the following trend in rates *k*_*ITO*/*nano*−*Au*/*PZT*_ > *k*_*ITO*/*PZT*/*nano*−*Au*/*PZT*_ > *k*_*ITO*/*nano*−*Au*._These results suggested that: (1) there are additional decay channels for the relaxation of the photo-excited density of states for nano-Au/PZT, and (2) nano-Au to PZT charge transfer yielded the accelerated relaxation of the bulk-like Au density of states. However, the photoinduced transmission on the poled ITO/PZT/nano-Au/PZT samples, i.e., at fields of +10 and −10 V, resulted in only minor changes in the relaxation dynamics. The conclusion suggested that the polarization at the PZT surface had little impact on the band bending and charge injection efficiency, in surprising contradiction to prior studies on ferroelectric-based solar cells. Independent characterization of the Schottky barrier heights could help elucidate their role in impacting the injection of hot carriers.

#### Hot Charge Injection From Au Nanoparticles to PbTiO_3_: Photochemical Reactions

**Figure 10:**
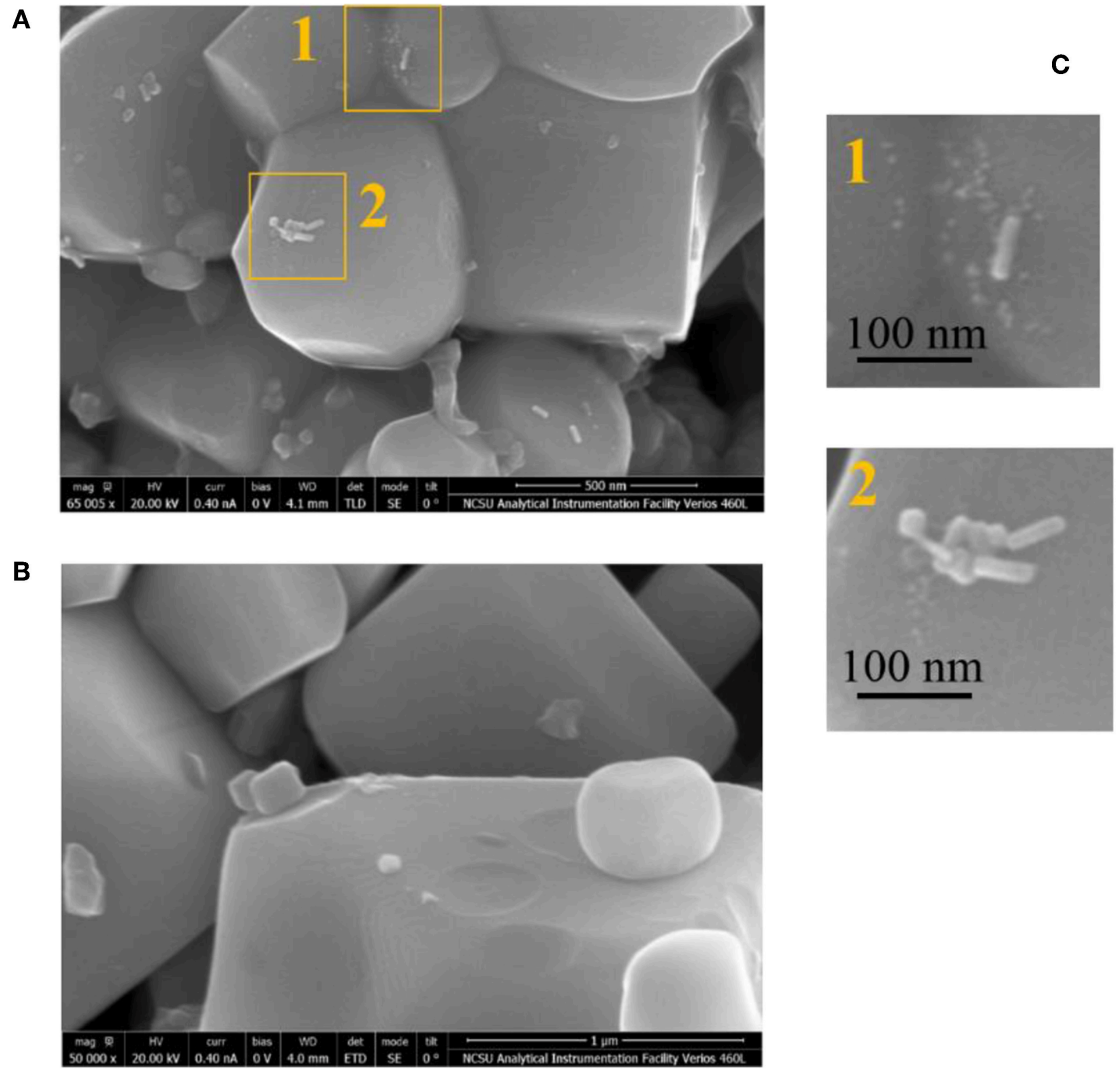
SEM images of PbTiO_3_ particles impregnated with and without AuNRs (**A,B**, respectively; Scale bar: 1 μm). The insets, 1 and 2 in **(C)**, show higher magnification SEM images of the PbTiO_3_ surfaces after the photochemical reduction of Pt driven by light excitation of the localized SPR of the AuNRs and hot carrier injection. The Pt islands are confirmed by EDS scanning and are visible as white dots near the deposited AuNRs to within ~100 nm on the surfaces. Figure adapted from Ortiz et al. (2018). Copyright 2018 American Chemical Society.

Direct evidence of hot carrier injection from metal to ferroelectric PbTiO_3_ (PT) particles was first reported by Ortiz et al. via the photochemical reduction of Pt on composite AuNRs/PT particles (Ortiz et al., 2018). The AuNR particles were synthesized as long nanorods with high aspect ratios of ~5:1 and impregnated onto micron-sized ferroelectric PT particles. The AuNRs/PT particles were suspended in an aqueous solution of H_2_PtCl_6_ with methanol as a sacrificial agent. The light-driven reaction proceeds by the photochemical reduction of the Pt(IV) cations in solution to form Pt(s) islands on the PT surfaces, together with the concomitant oxidation of methanol to carbon dioxide. The AuNRs/PT particles were irradiated by photons from 976 nm near IR light (NIR, 2.0 W/cm^2^), thus exciting the localized SPR and excluding the direct bandgap excitation of the ferroelectric PT. After irradiation under 976 nm light for 30 min, the AuNR/PT particles were then characterized by SEM to exhibit photochemically deposited Pt islands over the surfaces. The Pt islands were localized within a region about <100 nm from the AuNRs on the PT surfaces, as shown in Figure 10A (regions 1 and 2, see Figure 10C) and confirmed using EDX scans. As a control, no Pt islands were found to grow on the ferroelectric PT particles in the absence of AuNRs (Figure 10B). These results demonstrated that hot carrier injection occurs from the AuNR to ferroelectric particles, which drove the photochemical reduction of the Pt at the surfaces.

**Figure 11:**
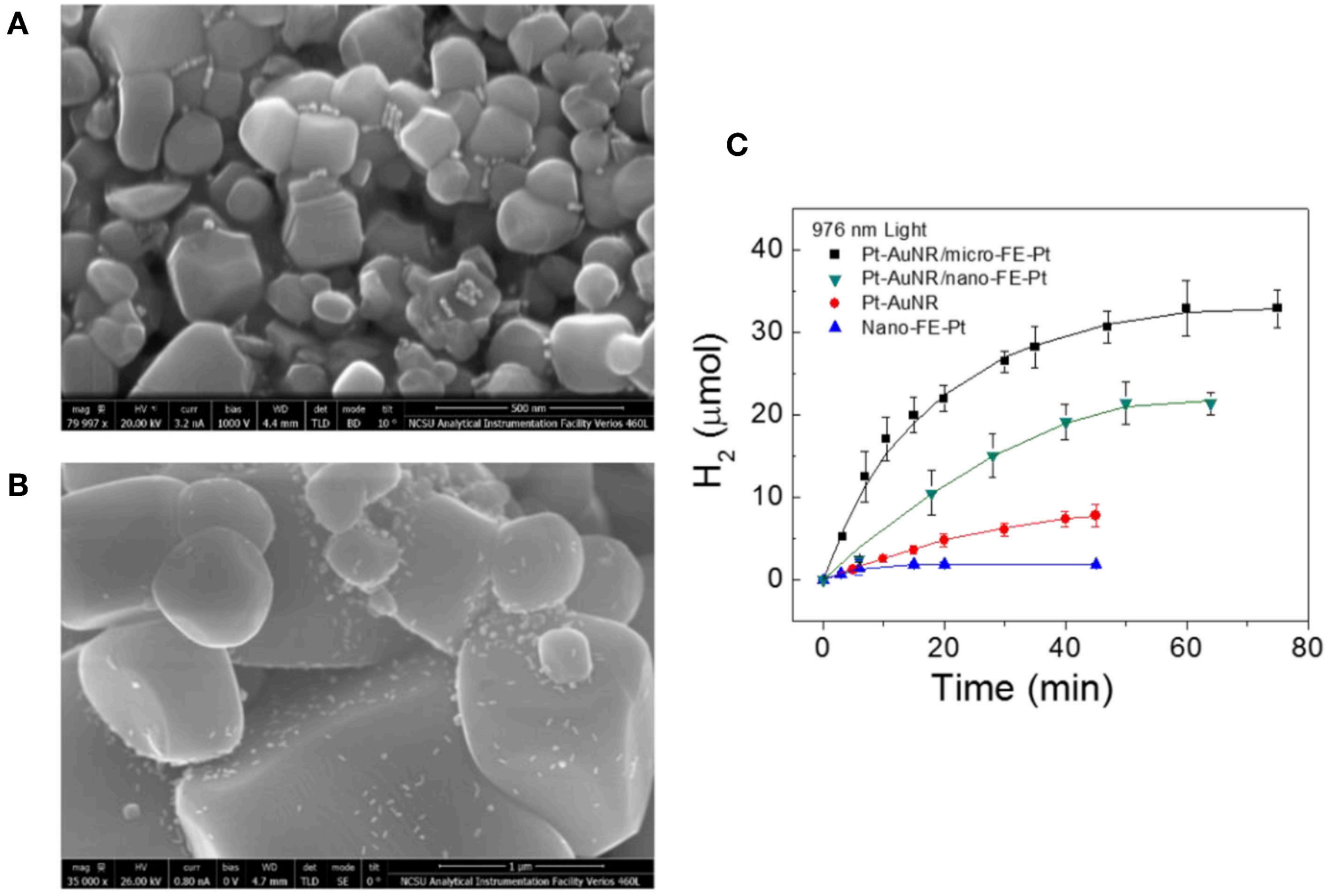
SEM images of ferroelectric PT particles impregnated with Pt-end-capped AuNRs, for the **(A)** nano-PT particles with dimensions of ~125 nm and for the **(B)** micro-PT particles with dimensions of ~1 μm. The AuNRs appear as white dots, with scale bars of 500 nm. **(C)** Hydrogen production vs. time for the Pt-AuNR/micro-FE-Pt and Pt-AuNR/nano-FE-Pt particles under near-IR irradiation. For comparison, photocatalytic hydrogen production for Pt-AuNRs and nano-PT particles are also plotted. Figure adapted from Ortiz et al. (2018). Copyright 2018 American Chemical Society.

As PbTiO_3_ is a known photocatalyst for water reduction when excited by bandgap light, it is anticipated that the hot carrier injection at the Au-PT interface should also lead to the light-driven production of molecular hydrogen at its surfaces, but with the use of photons lower than bandgap energies. In a study by Wang et al., the first example of water reduction to molecular hydrogen driven by hot carrier injection was demonstrated for the combination of Pt-end-capped AuNRs, i.e., Pt-AuNRs, and ferroelectric PT. Similar conditions were employed for the photochemical deposition of Pt, which involved irradiating the samples with near IR light at a wavelength of 976 nm and methanol as a sacrificial reagent. For the ferroelectric PT, both micron-sized (micro-PT; average diameter of ~1 μm) and nanosized (nano-PT; average diameter of ~125 nm) particles were used to determine the relative impacts of the surface area and loading with AuNRs. Shown in Figures 11A,B, the SEM images show that the AuNRs are distributed evenly over the nano-PT and micro-PT surfaces. The average loading of the AuNRs on the particles were ~0.60 (AuNRs:PT) for nano-PT and ~280 for 1 μm micro-PT. As the AuNRs are relatively sparsely deposited on the PT surfaces, the net “effective PT surface area,” i.e., the area beneath the AuNR and in its vicinity that the hot electrons can diffuse (~100 nm), is nearly a constant, enabling a direct comparison of the hydrogen production rates. Using sub-bandgap photons with a wavelength of 976 nm and a power density of 2.0 W cm^−1^, molecular hydrogen was produced with the AuNRs/PT particles at initial rates of 1.41 ± 0.40 μmol H_2_ min^−1^ for nano-PT particles and 0.88 ± 0.27 μmol H_2_ min^−1^ for micro-PT particles, as shown in Figure 11C. As controls, the ferroelectric PT particles alone showed little detectable activity, while the Pt-end-capped AuNRs alone exhibited a low initial photocatalytic rate of ~0.25 μmol H_2_ min^−1^. For equal amounts of plasmonic Au nanorods, the initial photocatalytic rates for hydrogen production were thus increased by a factor of ~5.6 when interfaced with the ferroelectric PT particles. A photon efficiency of up to ~0.28% for near-infrared light was measured, significantly higher than for previously reported metal-semiconductor combinations (0.01–0.1%) (Pu et al., 2013; Robatjazi et al., 2015).

The photocatalysis experiments have demonstrated that AuNRs can inject hot electrons into the ferroelectric particles upon irradiation of their localized SPR, and that the injected electrons are driving reduction reactions at the ferroelectric surfaces. It is notable that the interfaces between the plasmonic and the ferroelectric particles were not controlled in terms of positive or negative surface polarization. The natural randomization of the surface sites over which the AuNRs are deposited will lead to these making interfacial contact over a fraction of surfaces with suitable polarization directions, which favors hot electron injection over a Schottky barrier, i.e., the P(+) surfaces. Yet, a significant enhancement of the photocatalytic rates is found.

**Figure 12:**
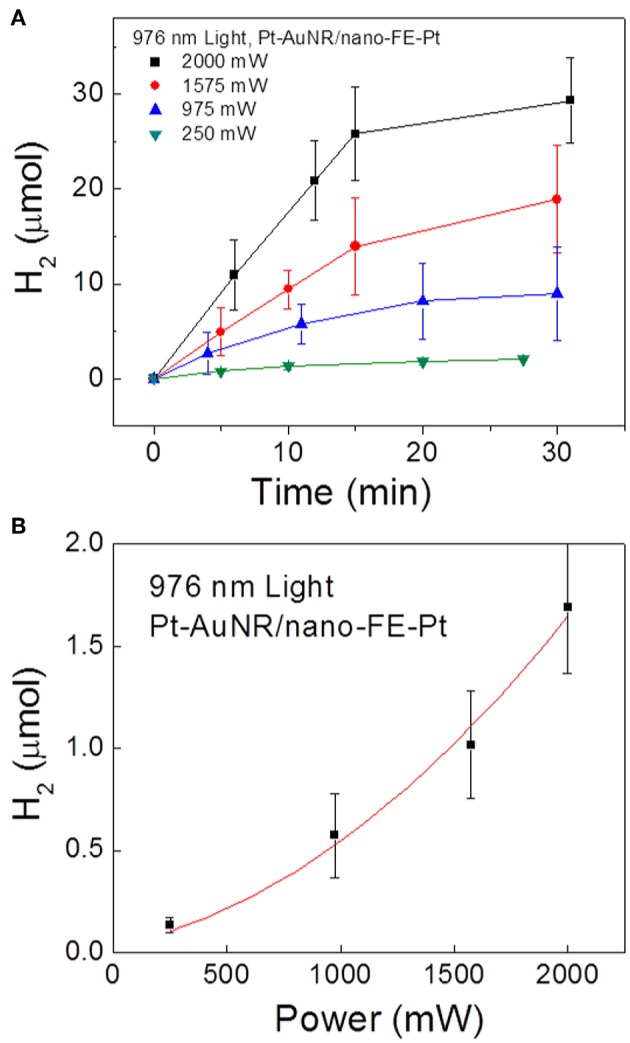
Photocatalytic H_2_ gas production on Pt-AuNR/nano-PT particles under 976 nm light at power densities of 250, 975, 1,575, and 2,000 mW. **(A)** Measured H_2_ production vs. time at the different irradiation powers, and **(B)** initial reaction rate vs. irradiation power. Figure adapted from Ortiz et al. (2018). Copyright 2018 American Chemical Society.

To further probe how the SPR-generated hot electrons are involved in the photocatalytic reaction, the initial hydrogen production rates were measured as a function of the near-IR irradiation power, plotted in Figure 12. Interestingly, a non-linearly increasing rate ~ power relationship is observed, in contrast to the linear relationship for the same reaction usually observed on metal-only particle surfaces. The non-linear relationship is possibly introduced during the charge injection process: only when the hot electrons have an energy higher than the interfacial energy barrier can they be transferred to ferroelectric particles and participate in the chemical reaction. The photo-excited electron energy, i.e., shown in Figure 1C, follows Fermi-Dirac distribution:

(8) $$\begin{array}{l} f\left(E\right)=\frac{1}{\exp\left[\left(E-E_{F}\right)/k_{B}T\right]+1} \end{array}$$

where *f* is the probability an energy level is filled, *E*_*F*_ is the Fermi level, *k*_*B*_ is the Boltzmann constant, and *T* is the effective temperature. Given that recent models show that the effective electron temperature increases linearly with respect to the fluency of the photon flux, the total number of “high energy” hot electrons exceeding a large threshold value increases non-linearly, as described previously (Manjavacas et al., 2014). This is consistent with the observed non-linear reaction rate ~ irradiation power relationship.

In these studies using Au nanoparticle interfaced with ferroelectrics, the Au nanoparticles were utilized to generate hot electrons because of their efficiency in harvesting solar energies down into the infrared wavelengths (Hartland, 2011; Ye et al., 2012; Jing et al., 2014; Kakekhani and Ismail-Beigi, 2016). The ferroelectrics, PT and PZT, were selected owing to their large and stable ferroelectric polarization, and they have been investigated in several prior studies that demonstrated a strong polarization-dependent modulation of the Schottky barrier height with the surface Au nanoparticles. For example, the ferroelectric PT has been characterized as an *n*-type semiconductor with a bandgap of ~2.7 eV (Reddy and Parida, 2013) and an electron affinity χ of ~3.5 eV (Pintilie et al., 2008; Li et al., 2012; Suriyaprakash et al., 2017). When Au (work function *Φ*_M_ = 5.1 eV) is in contact with a normal semiconductor with a similar electron affinity as that of PT, the semiconductor bands bend upward at their surface, leading to a low-energy Schottky barrier on the order of ~0.1–0.3 eV (Zhang and Yates, 2012; Pintilie et al., 2014). On the P(+) oriented surfaces of PT, the band bending is decreased or even reversed, while on the P(–) surfaces of PT the band bending will be further increased. It is conjectured that the lower energy barrier would favor the charge injection from Au to ferroelectric particles, leading to a higher efficiency in hot charge injection. In addition, it is also proposed that the surface polarization in the ferroelectric particles could trap the injected electrons at the domain boundaries (Wu et al., 2012; Sluka et al., 2013), which prevents them from flowing back to the metal (being quenched) and extending their lifetimes.

## Conclusions

In summary, the synergistic combination of plasmonic and ferroelectric particles represents a novel pathway for harnessing the efficient production and injection of hot carriers generated by the excitation of localized SPR. The energetic distribution of hot carrier generation in plasmonic nanoparticles is a sensitive function of the particle sizes and chemical compositions, and under optimal conditions, can result in the generation of hot carriers with energies that are from ~0.1 to 1.5 eV above and below the Fermi level. Concomitantly, a detailed understanding of the energetic structure at metal-ferroelectric interfaces is emerging and demonstrating that the modulation of the Schottky barrier height and band bending can be manipulated by the ferroelectric surface polarization. The Schottky barrier height, for example, typically varies from ~0.1 to 0.4 eV for common metal-ferroelectric interfaces and is thereby compatible with the energetic distribution of hot carrier generation in plasmonic nanoparticles. These synergistic features of their electronic properties have resulted in several novel studies involving the use of metal-ferroelectric interfaces for investigating switchable diode behavior as well as solar cells with high power conversion efficiencies. Most promisingly, these results show that by combining plasmonic and ferroelectric particles, novel photoelectrochemical and photocatalyst designs are possible for solar energy conversion.

## Future Prospects

Currently, the field of ferroelectric-interfaced plasmonic nanoparticles is still in its infancy, with only a very limited number of studies in this novel direction. Future investigations will aid in more deeply understanding the underlying mechanisms of hot electron generation and injection at metal-ferroelectric interfaces, designing more optimal integrated systems, and using these catalysts to efficiently drive photocatalytic reactions at the surfaces using solar energy. Among the prominent current challenges, the quantitative relationship between the charge injection/reaction rate and the ferroelectric polarization needs to be elucidated, such as via characterization of the band banding using I–V curves and X-ray photoelectron spectroscopy (XPS) (Chen and Klein, 2012). Second, the reactive sites, including both reductive and oxidative sites, need to be mapped out over these integrated systems. Third, the ferroelectric-plasmonic systems can be further tuned through changes in composition, e.g., in order to tune the bandgap of the ferroelectric or its surface polarization to facilitate changes in interfacial band-bending and charge injection. Fourth, the exposed facets of the ferroelectrics may play an important role in the Schottky barrier height. For example, facet-dependent photocatalytic activity is a common feature of photocatalyst particles, such as what has been reported for Cu_2_O (Yuan et al., 2016). Controlling the anisotropic growth of ferroelectric particles can also serve as a means in tuning the photocatalytic activity. Finally, alternative plasmonic materials can also be investigated which are less expensive, such as silver or copper. It can be anticipated that the optimization of interfacial metal-ferroelectric systems will offer remarkable new capabilities to achieve high solar conversion efficiencies at low-energy wavelengths, i.e., extending into the infrared wavelengths, and which is not possible in conventional semiconductor-based systems.

## Author Contributions

VK, PM, and GW wrote the manuscript. All authors have intellectual contributions to this work.

### Conflict of Interest Statement

The authors declare that the research was conducted in the absence of any commercial or financial relationships that could be construed as a potential conflict of interest.
